# Lactobacillus Modulates the Rumen Microbiota and Transcriptome to Enhance Nutrient Digestion in Yaks Fed High-Concentrate Diets During the Cold Season

**DOI:** 10.3390/ani16172644

**Published:** 2026-08-24

**Authors:** Hao Ren, Qian Chen, Majireding Aikelaimuc, Liang Qin, Guangfeng Zhang, Linlin Liu, Jianlei Jia

**Affiliations:** 1 School of Life Sciences, Qilu Normal University, Jinan 250200, China; renhao997@163.com (H.R.); chenqian910@sohu.com (Q.C.); 2 Teamgene (Shandong) Agricultural Technology Co., Ltd., Zibo 255086, China; 3 Taxkorgan Tajik Autonomous County Livestock Breeding Center, Kashi 845250, China; majierdingkashi@163.com; 4 Zhangqiu Animal Husbandry and Veterinary Science Development Center, Jinan 250200, China; qnld@sina.com (L.Q.); 15764147293@163.com (G.Z.); liulinlin919@163.com (L.L.)

**Keywords:** lactobacillus, Pamir yak, phenotypic data, multi-omics integration, high-concentrate diet

## Abstract

Intensive yak fattening in cold alpine regions requires essential long-term high-concentration feeding, which disrupts rumen microbial homeostasis and causes inefficient digestion of nutrients. Lactobacillus may have probiotic potential for ruminants, yet its regulation of rumen function is poorly understood under high-energy diets. During a 120-day feeding experiment, 120 male Pamir yaks were allocated to four dietary treatments, with LEG and LLG serving as the primary comparison for evaluating 0.02% Lactobacillus supplementation to explore the regulatory effects of Lactobacillus supplementation on the rumen microbiota and host metabolism of yaks during a fattening process based on a concentrate feed diet via phenotypic data (body wight and nutrient digestibility) and multi-omics analyses (rumen microbial sequencing and rumen epithelial transcriptome) in a concentrate-based rearing yak model. The results showed that Lactobacillus intervention reduced OTU (Operational Taxonomic Unit) richness during the early fattening period and subsequently promoted microbial recovery through colonization resistance, which significantly enhanced microbial diversity (*p* < 0.05), and restructured the microbial community structure toward efficient energy utilization under high-concentrate feeding by reducing Prevotellaceae and Ruminococcaceae abundance, increasing the Bacillota/Bacteroidota ratio (*p* < 0.05). Concurrently, Lactobacillus enhanced the apparent digestibility of dry matter, crude protein, and fibrous components (*p* < 0.05). According to the transcriptomic analysis, there was an activation of signaling pathways related to IL-18 and TNF, and up-regulation of immune-and metabolism-related genes, in addition to strengthening the rumen mucosal barrier function. Multi-omics integration supported that dietary supplementation of Lactobacillus can optimize rumen fermentation, enhance nutrient digestion, and strengthen immune defense in Pamir yaks fed high-concentrate in cold seasons. These modifications demonstrate the positive effects of the Lactobacillus supplementation strategy on yak rumen health without interfering with the high-energy intensive rearing pattern. The present research presents a scientific basis for the use of targeted probiotic strategies to improve the rumen health and efficiency of alpine yak production systems.

## Simple Summary

Feeding yaks on high-energy diets is common in cold Alpine regions, but these diets can upset the animals stomach microbes and digestion. Our research showed that Lactobacillus could help maintain yak health and productivity in Alpine farming systems. In a 120-day trial, 120 male Pamir yaks were assigned to four dietary treatments; the primary comparison evaluated a low-energy diet group (LEG) or with 0.02% Lactobacillus (LLG), while control feeding diet group (CKG) and a high-energy diet group (HEG) served as reference groups. The Lactobacillus first lowered microbial diversity in the rumen, but it later recovered and became even more diverse. It also improved digestion of dry matter, protein, and fiber, and boosted immune responses. Overall, Lactobacillus made the rumen work better, improving digestion and immunity in yaks on high-energy diets.

## 1. Introduction

The intensive feeding strategy focused on a high-concentrate diet has significantly enhanced the efficiency of livestock production [1]. However, it simultaneously elevates gastrointestinal metabolic pressures, presenting new challenges for animal welfare as well as the quality of milk and meat products. It is widely recognized that ruminants require between 40% and 70% of their diet to consist of roughage in order to support proper gastrointestinal tract (GIT) function and maintain a balanced microbiota [2]. In pursuit of greater economic benefits through improved daily weight gains, some livestock producers have increased the dietary concentrate proportion to over 75% [3,4]. Prolonged use of such diets can alter microbial metabolism, accelerate volatile fatty acid (VFA) accumulation, and lower rumen pH [5]. These changes might disturb microbial community dynamics and jeopardize the morphology of the intestinal epithelium [6]. Because rumen structure and function are closely related to health and productive performance [7], the consequences of feeding management, diet composition, and microbial ecology must be considered together [8,9]. Therefore, developing a sustainable feeding system for domestic yaks that balances efficient energy intake with rumen health is a crucial strategy for promoting their effective management.

The ruminant gastrointestinal microbiome is dynamic and responds to age, physiological development, and dietary composition [10]. The fattening period is one of the critical stages during which changes in microbiota can significantly impact host health or product quality [11]. Some previous studies suggest that rumen microorganisms play a crucial role in shaping animal health and host phenotypes into adulthood. For instance, adding corn bran to cattle diets alters the ratio of Firmicutes to Bacteroidetes, thereby regulating papillae morphology and promoting rumen epithelial growth while enhancing microbial diversity and the organism’s anti-inflammatory response [12]. Similarly, incorporating caragana into sheep diets modifies the composition of gut butyrate-producing bacteria, improving meat tenderness and fatty acid content [13]. Importantly, studies have shown that increasing concentrate levels has been associated with lower relative abundance of dominant rumen taxa [14,15]. High-concentrate diets (HCDs) also reshape short-chain fatty acid (SCFA) profiles and host metabolism, including epithelial expression of SCFA transporters and the acetate-to-propionate ratio [16]. Additionally, previous studies have confirmed that microbial activity generates more than 60% of rumen fermentation metabolites and can modify polyunsaturated fatty acid metabolism in ways that affect HCD-associated weight gain [17]. Nevertheless, there remains a lack of comprehensive insights into the adverse effects of HCD on gut microbiota dynamics and metabolic functions, particularly in yaks.

In contemporary systems of feeding and managing ruminants, adding probiotics or prebiotics during the rearing of animals has been demonstrated to enhance both the structural and functional integrity of the rumen microbiota, boost host immunity and disease resistance, and improve the quality of products derived from animals [18,19]. Recent research indicates that probiotics can help restore the microbial homeostasis of the gastrointestinal tract (GIT) and provide beneficial short-chain fatty acids (SCFAs) [20,21]. Evidence suggests that Lactobacillus is among the most significant beneficial bacteria found in the ruminant rumen, offering a broad range of physiological advantages [22]. These advantages encompass the modulation of gastrointestinal microbiota, stimulation of peristalsis, maintenance of the micro-ecological balance, enhancement of gut functionality as a whole, improved efficiency in feed utilization, and support for the production of healthy animals. Moreover, species of Lactobacillus are considered non-toxic, biodegradable, and biocompatible, rendering them viable alternatives to traditional probiotic formulations [23]. Investigations have shown that many metabolites produced by Lactobacillus play a direct role in host metabolic pathways [24]; these substances function as detoxifying agents by facilitating the biohydrogenation of dietary polyunsaturated fatty acids (PUFAs) via the generation of antimicrobial peptides and SCFAs. This mechanism extends the residence time of probiotics within the rumen, decreases the population of aerobic bacteria present in the rumen microbiota, and thus enhances the efficiency of ruminal fermentation [25]. Despite this general agreement, it remains uncertain how extensively the components of exogenous probiotic microbiomes are integrated into the native rumen microbiome and what specific functional roles they fulfill within this intricate ecosystem.

The Pamir yak, an ideal model for studying the intervention of Lactobacillus in Microbiota–Host Cross-Talk, is a unique domesticated species known for its gastrointestinal tract adaptability in the Pamir Plateau region. This species efficiently exploits alpine grassland resources under the extreme environmental conditions of the plateau, which include hypoxia, severe cold, intense ultraviolet radiation (UVR), and food scarcity [26]. In this study, we collected monthly data on rumen liquid and body weight from experimental Pamir yaks, along with apparent traits associated with host nutrient digestion, between 1 November 2023 and 18 March 2024. During this period, the yaks were randomly divided into two groups: a concentrate-based rearing with a low-energy diet group (LEG) and a low-energy diet with Lactobacillus intervention group (LLG). Additionally, a control feeding diet group (CKG) and a high-energy diet group (HEG) were concurrently employed as comparative groups within a rigorously controlled experimental design. We comprehensively analyzed the structural and functional changes in the microbiome with and without Lactobacillus intervention during the intensive finishing period to explore the regulatory effects of Lactobacillus supplementation on host nutrient digestion, including yak digestion, microbiome composition, and transcriptome profiling during the fattening period.

## 2. Materials and Methods

All procedures were approved by the Animal Care Committee of Qilu Normal University, China (approval no. 20230361-109). Animal slaughter complied with the applicable national regulations for experimental animal slaughter and quarantine.

### 2.1. Animal Models and Experimental Design

The experiment was conducted at Junmahong Livestock Farm in Taxkorgan Tajik Autonomous County, Xinjiang, China. A total of 120 male Pamir yaks (4–5 years old; 250–280 kg) that had grazed native Pamir Plateau pasture were housed in roofed sheds for intensive feeding. The 140-day study ran from 1 November 2023 to 18 March 2024 and comprised a 20-day adaptation period, during which concentrate inclusion was gradually increased, followed by 120 days of data collection. Diets were formulated from estimated grazing intake and the Chinese Beef Cattle Raising Standard (NY/T 815-2004) [27], with a concentrate-to-forage ratio of 70:30 during the adaptation period and gradually adjusted to 50:50 during the data collection period. The requisite concentrated feeds for the experiment were manufactured by Teamgene (Shandong) Agricultural Technology Co., Ltd.(Zibo, Shandong, China), and the nutrient levels are shown in Table 1. The nutrient composition of the mixed diet was ascertained by the laboratory at Qilu Normal University by employing “Feed Analysis and Quality Test Technology”.

Yaks were stratified by body weight and randomly allocated with the SAS 9.4 simple random sampling procedure to four treatments (six sheds per treatment; five yaks per shed, with the shed as the experimental repetition, and each yak as an experimental unit). The LEG received a normal-energy diet (NEG 2.12 MJ/kg; 5 kg mixed ration/yak/day). The LLG received the same diet plus 0.02% Lactobacillus powder throughout the trial. The HEG received a high-energy diet (NEG 2.69 MJ/kg; 5 kg/yak/day), whereas the CKG received the traditional ultra-low-energy control diet used during the nutrient-deficient cold season (NEG 0.82 MJ/kg; 5 kg/yak/day). The commercial product contained Lactobacillus fermentum (1 × 10^9^ CFU/g; Wei’er Bioengineering Co., Ltd., Taian, China).

### 2.2. Sample Collection and Measurement

Body weight and one rumen-fluid sample were obtained from each yak before morning feeding on days 1, 30, 60, 90, and 120. Approximately 100 mL of rumen fluid was collected by stomach tube, filtered through four layers of gauze, and measured immediately for pH (PHS-3C, Shanghai, China). Samples were centrifuged at 350× *g* for 15 min at 4 °C, divided into two aliquots, and stored at −80 °C for 16S rRNA gene sequencing. Initial body weight, monthly fattening-stage weights (FSW1-FSW4), final body weight, and total average daily gain (TADG, g/day) were recorded or calculated.

During the final week, after 3 days of adaptation to individual metabolism cages, feces were collected from three LEG yaks and three LLG yaks. Feed and approximately 5% of the daily fecal output were sampled before 10:00 Beijing time. Daily fecal samples were pooled, acidified with 10% sulfuric acid to limit nitrogen loss, and stored at −80 °C by employing “Feed Analysis and Quality Test Technology”. Feed and feces were analyzed for dry matter (DM), ash, crude protein (CP), neutral detergent fiber (NDF), and acid detergent fiber (ADF) in the Qilu Normal University laboratory using standard ruminant-nutrition procedures.

Microbial DNA was extracted from rumen fluid and processed for 16S rRNA gene sequencing by Majorbio Co., Ltd. (Shanghai, China). DNA quality and concentration were checked by 1% agarose gel electrophoresis. The V3-V4 region was amplified with primers 5′-CCTACGGGNGGCWGCAG-3′ and 5′-GGACTACHVGGGTATCTAAT-3′, and libraries were sequenced on an Illumina NovaSeq/HiSeq X Ten platform. PCR comprised 2 min at 95 °C, 35 cycles of 95 °C for 2 min and 72 °C for 30 s, and a final extension at 72 °C for 5 min. A total of 16S rRNA data were processed with R 3.1.2, QIIME 1.9.1, and UPARSE. Reads were clustered into OTUs at 97% sequence identity and taxonomically assigned with the RDP Classifier 2.2. Alpha diversity was evaluated using rank-abundance and rarefaction curves and the Shannon, Chao1, Simpson, and ACE indices. Weighted UniFrac distances were used for PCoA and UPGMA analyses, and PICRUSt was applied for functional prediction. Taxa were compared with Wilcoxon rank-sum tests; FDR-adjusted *p* < 0.05 was considered significant.

Six experimental yaks from each group were randomly selected and humanely euthanized using a captive bolt pistol (positioned at the forehead and fired to achieve loss of consciousness), followed by immediate carotid exsanguination to ensure death. Immediately thereafter, four tissue specimens were separately collected from the dorsal and ventral regions of the rumen. Rumen tissue samples were transferred into 2 mL centrifuge tubes and stored at −80 °C for subsequent transcriptome sequencing by Majorbio Co., Ltd. (Shanghai, China). The RNA-seq library construction proceeded as follows: Total RNA was extracted from the samples and treated with DNase to remove genomic DNA contamination. mRNA was then enriched using oligo(dT)-conjugated magnetic beads. Following fragmentation, double-stranded cDNA was synthesized using hexamer random primers. Library construction was completed through end repair, A-tailing, and adapter ligation. Libraries were quality-assessed using the Agilent 2100 Bioanalyzer (Agilent Technologies, Waldbronn, Germany), and high-throughput sequencing was performed to generate raw reads, followed by removal of adapter sequences and low-quality reads. Clean reads were quality-filtered (Q30, GC content) and aligned to the reference genome using Hisat2 v2.1.0. Gene-level counts were obtained with htseq-count v0.11.2 and normalized to FPKM for expression quantification, followed by PCA. Differential expression analysis was performed using DESeq2 v1.22.2 (negative binomial test), with significance thresholds of q < 0.05 and |fold change| > 2. Differentially expressed genes were subsequently subjected to GO and KEGG pathway enrichment analyses.

### 2.3. Statistical Analysis

Data were screened for outliers with box plots and tested for normality with the Shapiro–Wilk test. Homogeneity of variances was assessed using Bartlett’s test (normally distributed data) or Levene’s test (non-normally distributed data). Results are reported as mean ± SEM and were analyzed under a completely randomized design in SAS 9.4. To identify significant differences among groups, normally distributed data were compared with the Duncan’s multiple range test (DMRT) procedure; otherwise, the Kruskal–Wallis test was used. *p* < 0.05 indicated significance and *p* < 0.10 a trend.

## 3. Results

### 3.1. Apparent Traits, Rumen Fermentation, and Microbial Community Parameters in Yak

All Yaks received no antibiotics and exhibited no mortality throughout the finishing period. The weight gain of LLG yaks was consistently higher than that of LEG yaks except for the first month of the fattening stage (Table 2). Lactobacillus intervention had a significant effect on yak final body weight (FBW) and fattening stage weight (FSW) (*p* < 0.05), particularly during the third and fourth months of the fattening stage. Although Lactobacillus intervention did not significantly affect total average daily gain (TADG), a significant growth trend was observed. FSW increased significantly with the progression of the finishing period (*p* < 0.05); however, a temporary decline in FSW1 and FSW2 was noted during the first two months of the experiment, which coincided with changes in gut microbiota composition as the yaks transitioned from a normal-energy diet to a Lactobacillus-intervention diet.

The specialized rumen digestive system of ruminants creates essential divergence in feed digestion efficiency and metabolic pathways compared to non-ruminant mammals. The rumen harbors a diverse, abundant, and stable microbial community, which maintains pH within a suitable range, creating optimal conditions for feed digestion and various microbial metabolisms. As presented in Table 3, Lactobacillus supplementation significantly improved the apparent digestibility of key nutrients, particularly dry matter, crude protein, and fiber fractions. The LLG exhibited significantly higher digestibility of dry matter, crude protein, and fibrous components (NDF, ADF, CF) compared to the LEG group (*p* < 0.05). The CP digestibility increased from 41.45% in the low-energy group to 49.97% in the high-energy group, while no significant differences were observed for EE or ash content among groups (*p* > 0.05). The result showed that Lactobacillus supplementation often results in fermentation function of the rumen microbiota caused by high-concentrate diets, which plays a pivotal role in breaking down complex food components such as dietary fiber and regulating host functional homeostasis through interactions involving cellulose and amino acids.

### 3.2. Overview of Changes in Experimental Yak Microbiota Composition over the 120 Days of the Experimental Period

**Figure 1:**
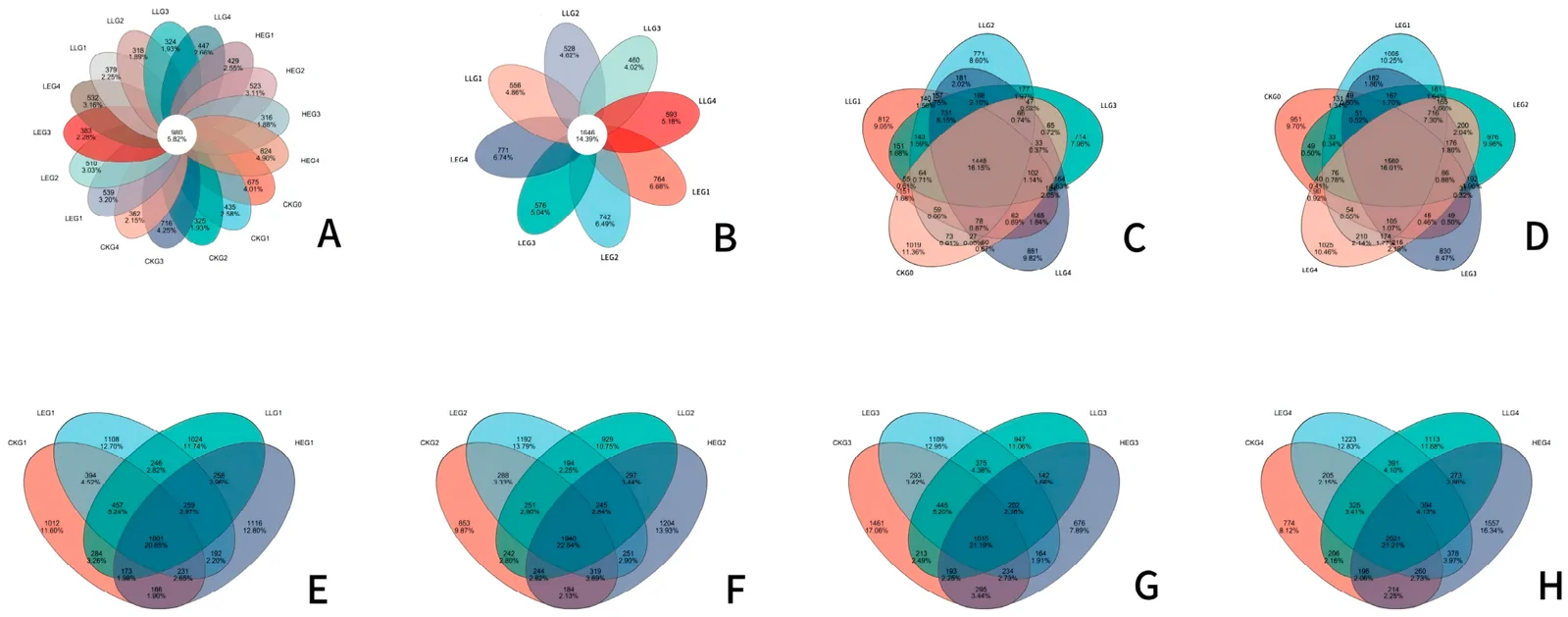
Venn diagrams of rumen fluid at different fattening stages in yaks fed diets containing CNK (0.11 MJ/kg NEG), LEG (2.12 MJ/kg NEG), LLG (2.12 MJ/kg NEG + 0.02% Lactobacillus), and HEG (2.69 MJ/kg NEG). (**A**) Venn diagram of all groups during the fattening period; (**B**) Venn diagram of experimental groups during the fattening period; (**C**) Venn diagram of LLG group during the fattening period; (**D**) Venn diagram of LEG group during the fattening period; (**E**) Venn diagram of all groups for the first month of fattening; (**F**) Venn diagram of all groups for the second month of fattening; (**G**) Venn diagram of all groups for the third month of fattening; (**H**) Venn diagram of all groups for the fourth month of fattening.

In the analysis of the species Venn diagram, by comparing the microbial composition at the OTU level among different treatment groups, 16,882 OTUs were identified corresponding to 23 phyla, 46 classes, 114 orders, 235 families, 644 genera, and 1292 species. The total number of shared OTUs among all groups was 980, accounting for 5.82% of the total OTUs. The control group (CKG0) had 675 unique OTUs, representing 4.01% of the total OTUs, while the experimental groups (LEG1-HEG4) had unique OTUs ranging from 316 (1.88%) to 824 (4.90%). These findings suggest that dietary energy and Lactobacillus intervention exert both common regulatory effects and specific influences on the composition of the intestinal microbiota in yaks (Figure 1A). Post-Lactobacillus intervention, the average number of OTU reads was lower than that in LEG (Figure 1B). With the persistent intervention of Lactobacillus, the quantity of OTUs in the intestinal microbiota initially declined, particularly in the second and third months. Under continued supplementation, OTU numbers declined during months 2 and 3 but recovered in month 4 (Figure 1C,D), suggesting temporal reorganization of the resident community after supplementation. The variations in OTUs in each group throughout the monthly fattening process suggest that Lactobacillus may reduce the diversity of gut microbiota through multiple mechanisms, including substrate limitation, environmental modification, and colonization resistance among microorganisms within the host (Figure 1E–H).

**Figure 2:**
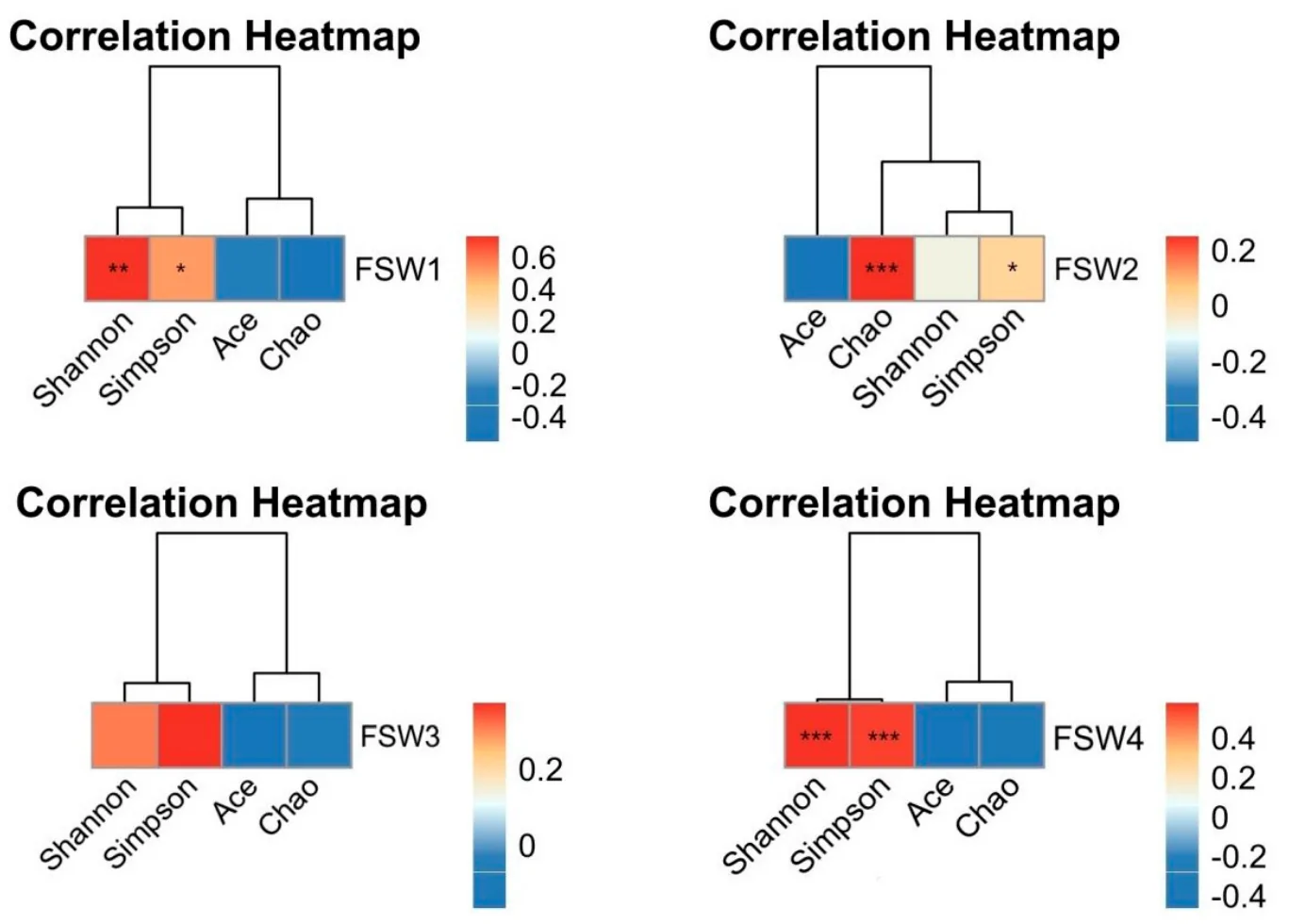
The correlation analysis between monthly weight gain and α-diversity indices. * significant correlation between LEG and LLG (*p* < 0.05), ** significant correlation between LEG and LLG (*p* < 0.01), and *** significant correlation between LEG and LLG (*p* < 0.001).

**Figure 3:**
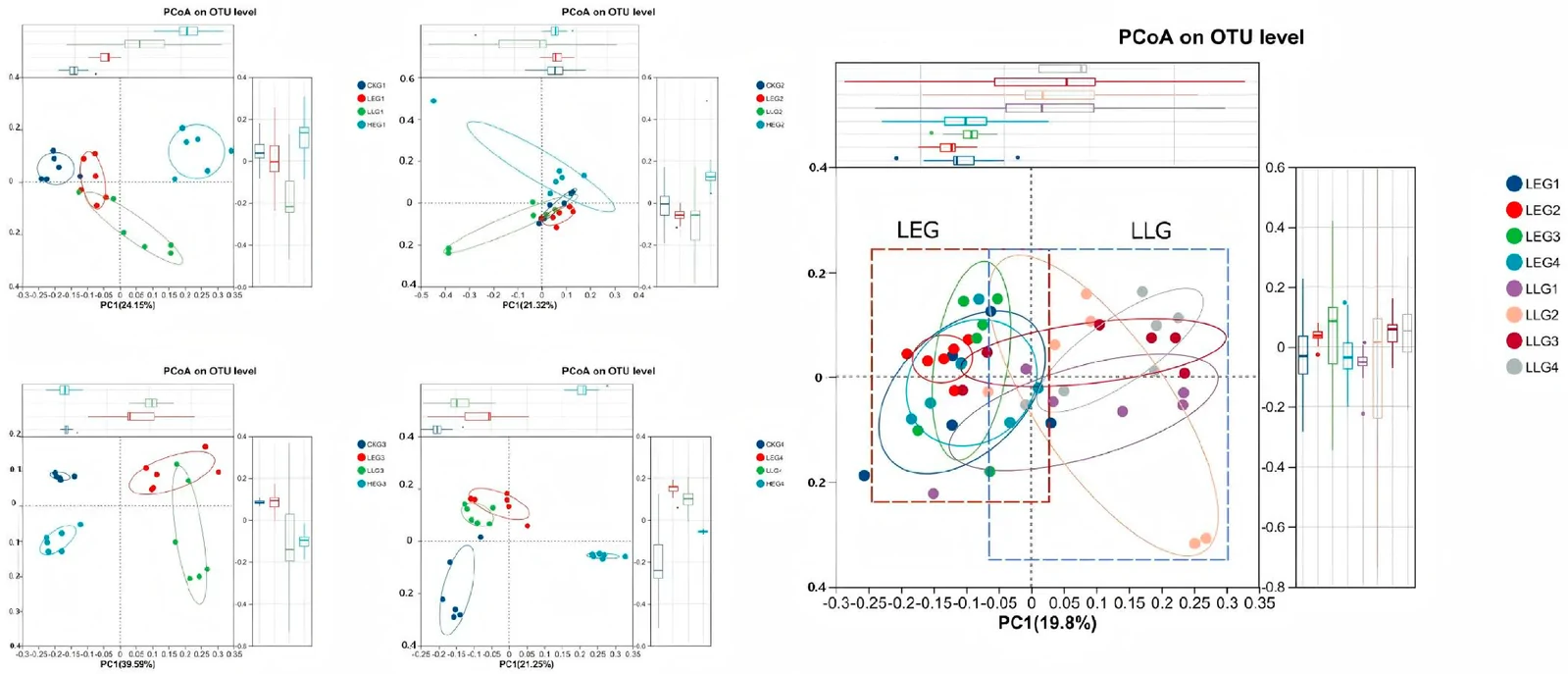
The β-diversity of rumen fluid at different fattening stages in yaks fed diets containing CNK (0.11 MJ/kg NEG), LEG (2.12 MJ/kg NEG), LLG (2.12 MJ/kg NEG + 0.02% Lactobacillus), and HEG (2.69 MJ/kg NEG).

Considering the influence of Lactobacillus intervention and a high-energy diet on the gut microbiome, we analyzed the structure and differences in the gut microbiota during different finishing periods. The α-diversity of the microbiome was estimated using the Chao, ACE, Shannon, and Simpson indices (Table 4). In the last two months, all α-diversity indices, including the Chao, ACE, Simpson, and Shannon indices of yaks, showed significant differences. The Shannon and Simpson indices of the high-energy group were lower than those of the low-energy group (*p* < 0.05), while the ACE and Chao indices exhibited an opposite trend (*p* < 0.05). Significant differences were observed in the Chao, ACE, Simpson, and Shannon indices between the LEG and LLG during different finishing periods. Specifically, the Simpson and Shannon indices of LLG were greater than those of the LEG (*p* < 0.05), whereas the ACE and Chao indices were lower (*p* < 0.05). Meanwhile, we performed a correlation analysis between monthly weight gain and α-diversity indices (Figure 2). The results indicated that the Shannon and Simpson indices had a significant effect on monthly weight gain, particularly in the 1st and 4th months. While the supplementation of Lactobacillus might slightly decrease the abundance of the microbial community, it significantly enhances the Shannon and Simpson diversity indices. Consequently, this improvement in microbial evenness contributes to greater community stability, thereby promoting nutrient metabolism and immune regulation. To further elucidate the influence of Lactobacillus intervention on the rumen microbial community of Pamir yaks, a principal coordinate analysis (PCoA) was performed on the gut microbiota composition of Pamir yaks using Weighted Unifrac (Figure 3). The results of the first principal component (PC1: 21.25% to 39.59%) and the second principal component (PC2: 12.03% to 14.67%) suggest that the different treatments exerted a certain influence on the microbial community during the fattening stage. By observing the distances among different groups (PC1: 19.8% and PC2: 11.88%), it can be discerned that the Lactobacillus intervention has a certain impact on rumen microbial community composition; specifically, the LEG was grouped together, while the LLG formed another cluster. Interestingly, we observed that during the fattening process, particularly after a one-month adaptation period, the bacteria Treponema and Alistipes were positively correlated with body weight gain. In contrast, Monoglobus, [Eubacterium]_coprostanoligenes_group, Ruminococcaceae, and Akkermansia were negatively correlated with body weight gain.

**Figure 4:**
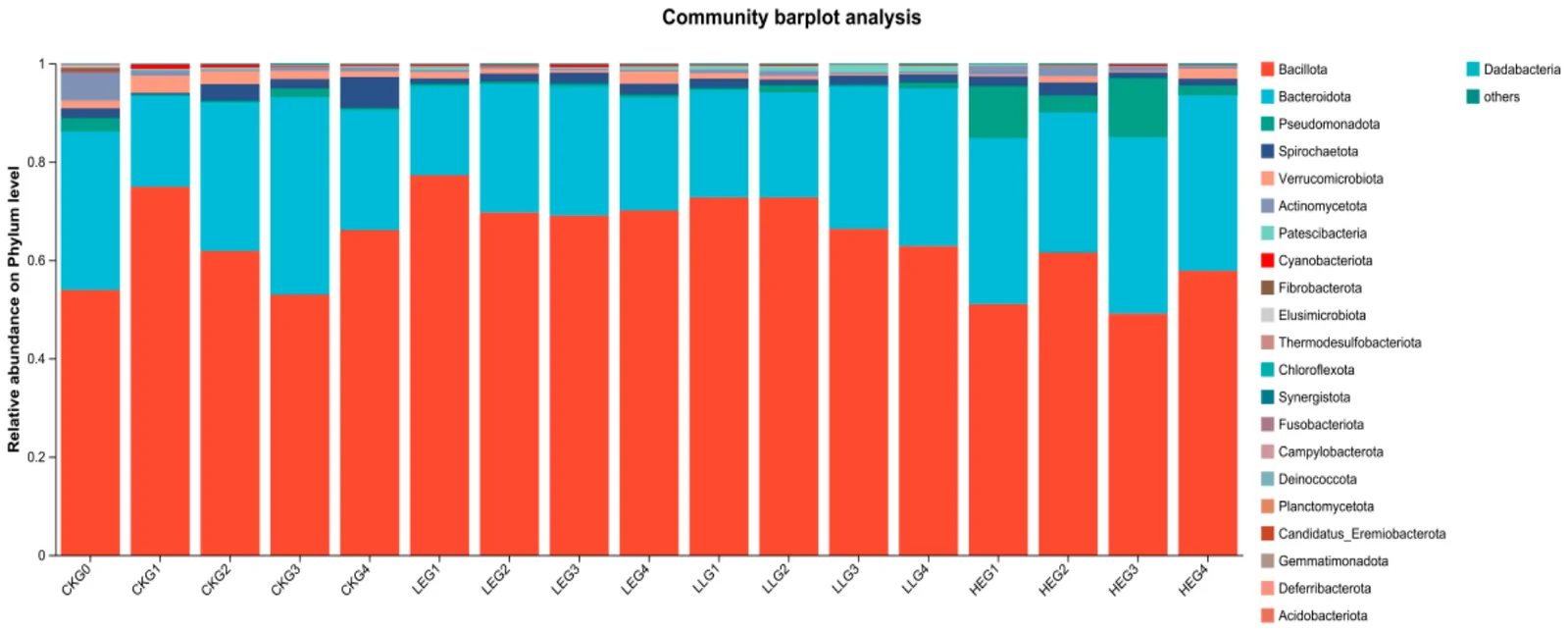
The distribution of rumen fluid microbial flora (genus level) at different stages in yaks fed diets containing CNK (0.11 MJ/kg NEG), LEG (2.12 MJ/kg NEG), LLG (2.12 MJ/kg NEG + 0.02% Lactobacillus), and HEG (2.69 MJ/kg NEG). Note. Values are presented as mean ± SEM (n = 6). Homogeneity of variances was confirmed by Levene’s test (*p* > 0.05).

At the phylum level (Figure 4), yak gut microbiota was dominated by Bacillota (53.89%) and Bacteroidota (32.26%) across all time points. Bacillota average abundance significantly increased throughout the treatment groups, from an average of 58.81% in the control groups (CKG and HEG) to 70.08% in the experimental groups (LEG and LLG), becoming the most dominant phylum. Bacteroidota reduced in abundance dramatically between thev control groups (average 31.04%) and experimental groups (average 24.72%). Post-Lactobacillus intervention, the average abundance of Bacillota decreased steadily between the LEG (average 81.29%) and the LLG (average 68.66%), and the abundance of Bacteroidetes fluctuated between the LLG and the LEG (average 26.05 and 13.82%, respectively). However, the elevated Bacillota (Firmicutes)/Bacteroidetes ratio (F/B) in the LLG was potentially closely correlated with the colonization of bacteria related to efficient fiber decomposition and the synthesis capacity of short-chain fatty acids, which might be attributed to the supplementation of Lactobacillus. Moreover, to a greater extent, Actinomycetota in the LLG were greater than in the LEG (*p* < 0.05), which demonstrated that, post-Lactobacillus intervention, the abundances of the microbiota in the yak gut related to fiber decomposition, energy supply, the nitrogen cycle, and immune regulation were higher than those of the LEG group.

**Figure 5:**
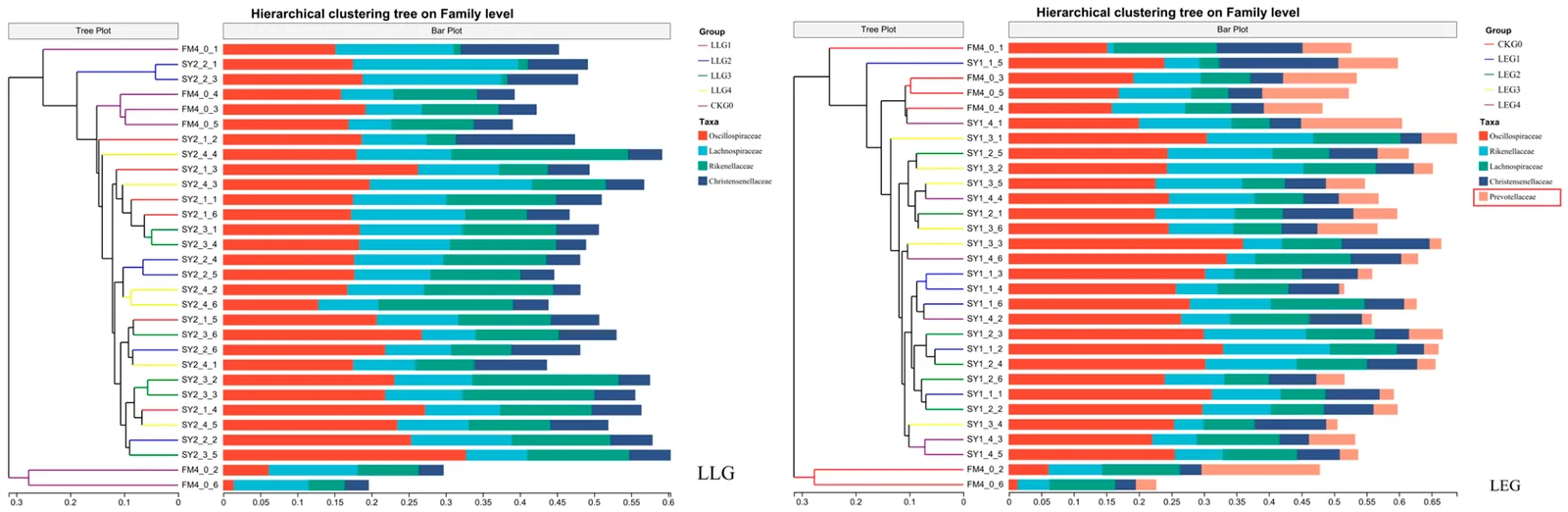
The mean relative abundance of rumen fluid microbial flora (family level) at different fattening stages in yaks fed diets containing CNK (0.11 MJ/kg NEG), LEG (2.12 MJ/kg NEG), LLG (2.12 MJ/kg NEG + 0.02% Lactobacillus), and HEG (2.69 MJ/kg NEG).

**Figure 6:**
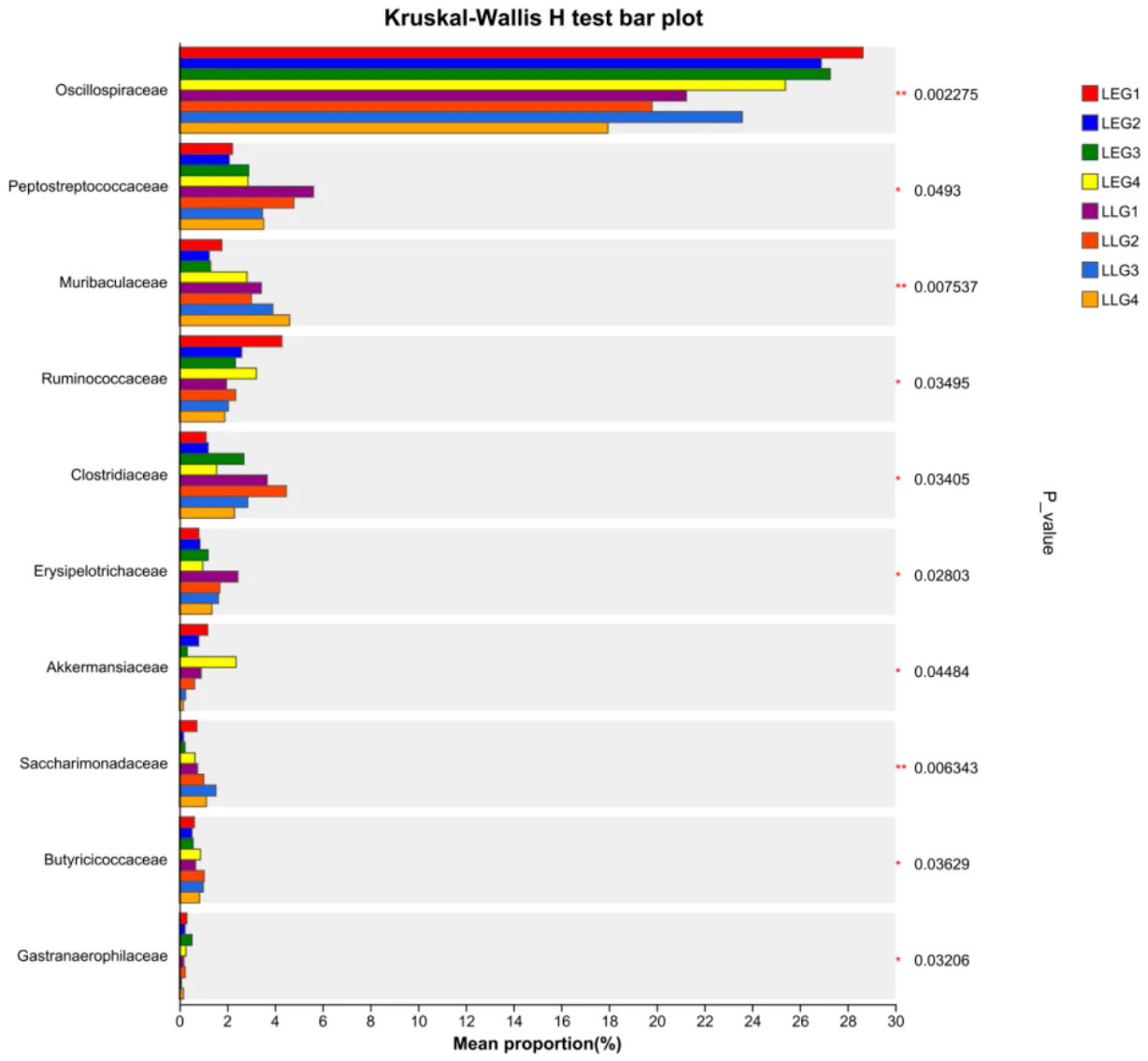
The enrichment taxa mean relative abundance of rumen fluid microbial flora (family level) analysis (Kruskal-Walis H test, *p* < 0.05) at different fattening stages in yaks fed diets containing CNK (0.11 MJ/kg NEG), LEG (2.12 MJ/kg NEG), LLG (2.12 MJ/kg NEG + 0.02% Lactobacillus), and HEG (2.69 MJ/kg NEG). * significant correlation between LEG and LLG (*p* < 0.05), ** significant correlation between LEG and LLG (*p* < 0.01).

**Figure 7:**
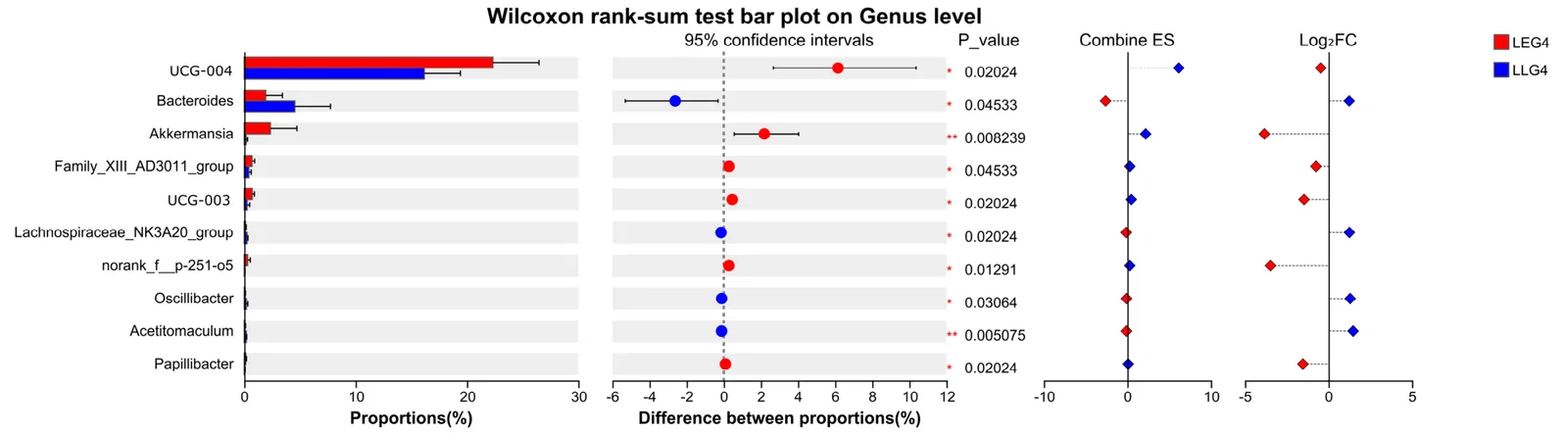
The enrichment taxa’s mean relative abundance of rumen fluid microbial flora analysis (genus level) (Wilcoxon rank-sum test, *p* < 0.05) at different fattening stages in yaks fed diets containing LEG (2.12 MJ/kg NEG) and LLG (2.12 MJ/kg NEG + 0.02% Lactobacillus). * significant correlation between LEG and LLG (*p* < 0.05), ** significant correlation between LEG and LLG (*p* < 0.01).

**Figure 8:**
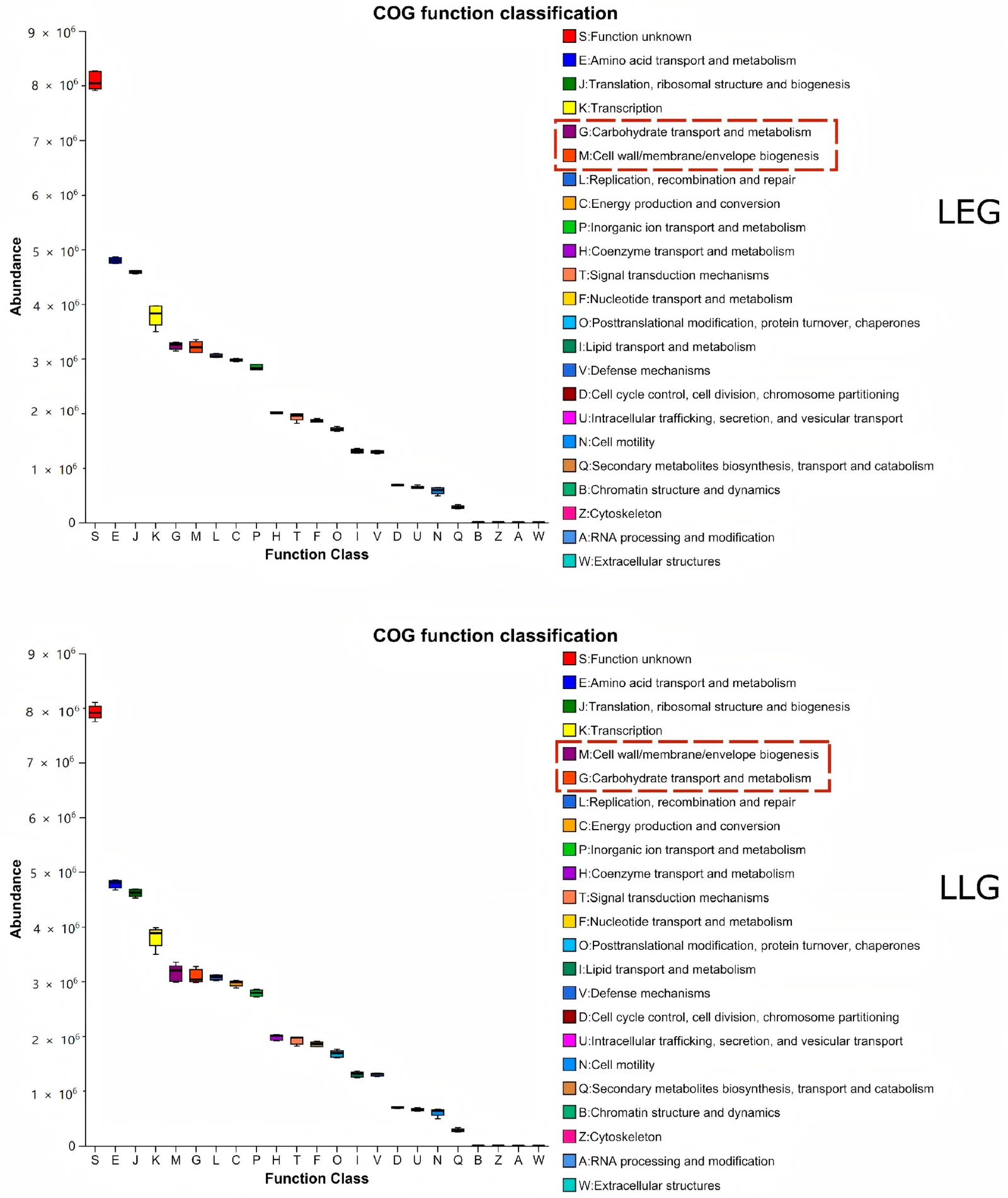
The COG analysis of rumen fluid microbial flora in yaks fed diets containing LEG (2.12 MJ/kg NEG) and LLG (2.12 MJ/kg NEG + 0.02% Lactobacillus).

The mean relative abundance of several taxa changed with the Lactobacillus supplement in yak intensive feeding mode based on a high-concentrate diet (Figure 5). Interestingly, yak fecal microbiota was dominated by Oscillospiraceae, Rikenellaceae, Lachnosporaceae, Christensenellaceae and Prevotellaceae at all time points of the LEG, while Prevotellaceae abundance experienced a significant reduction with Lactobacillus intervention (Figure 6), the average abundance of Oscillospiraceae (*p* = 0.0023), Ruminococcaceae (*p* = 0.0349), Akkermansiaceae (*p* = 0.0448) and Gastranaerophilaceae (*p* = 0.0321) reduced significantly with Lactobacillus intervention throughout the finishing period. The average abundance of Peptostreptococcaceae (*p* = 0.0493), Muribaculaceae (*p* = 0.0075), Clostridiaceae (*p* = 0.0431), Erysipelotrichaceae (*p* = 0.0281), Saccharimonadaceae (*p* = 0.0063) and Butyricicoccaceae (*p* = 0.0363) showed an increased tendency. At the genus level, UCG-005 (17.82%), Rikenellaceae_RC9_gut_group (11.61%), and Christensenellaceae_R-7_group (6.43%) were the dominant genera throughout the study. Further analysis revealed that Prevotellaceae_UCG-004 (*p* = 0.0241) and Prevotellaceae_UCG-003 (*p* = 0.0241) were significantly reduced after Lactobacillus intervention, and it was demonstrated that Lactobacillus acidifies the intestinal environment through lactic acid production, while the optimal growth pH of Prevotellaceae is typically close to neutral, leading to the inhibition of its metabolic activity with the reconfiguration of the microbial community structure (Figure 7). The results of PICRUSt-based COG prediction indicated a lower predicted representation of functions related to cell wall/membrane/envelope biogenesis and a higher predicted representation of functions related to carbohydrate transport and metabolism in LLG, which is consistent with the results of species difference analysis (Figure 8). After the addition of lactic acid bacteria, the degradation efficiency of dietary fiber was improved, thereby enhancing the digestion efficiency of nutrients.

### 3.3. Lactobacillus Re-Engineered High-Concentrate Diets Mediated Specific Transcriptional Expression Patterns in Yak Rumen

**Figure 9:**
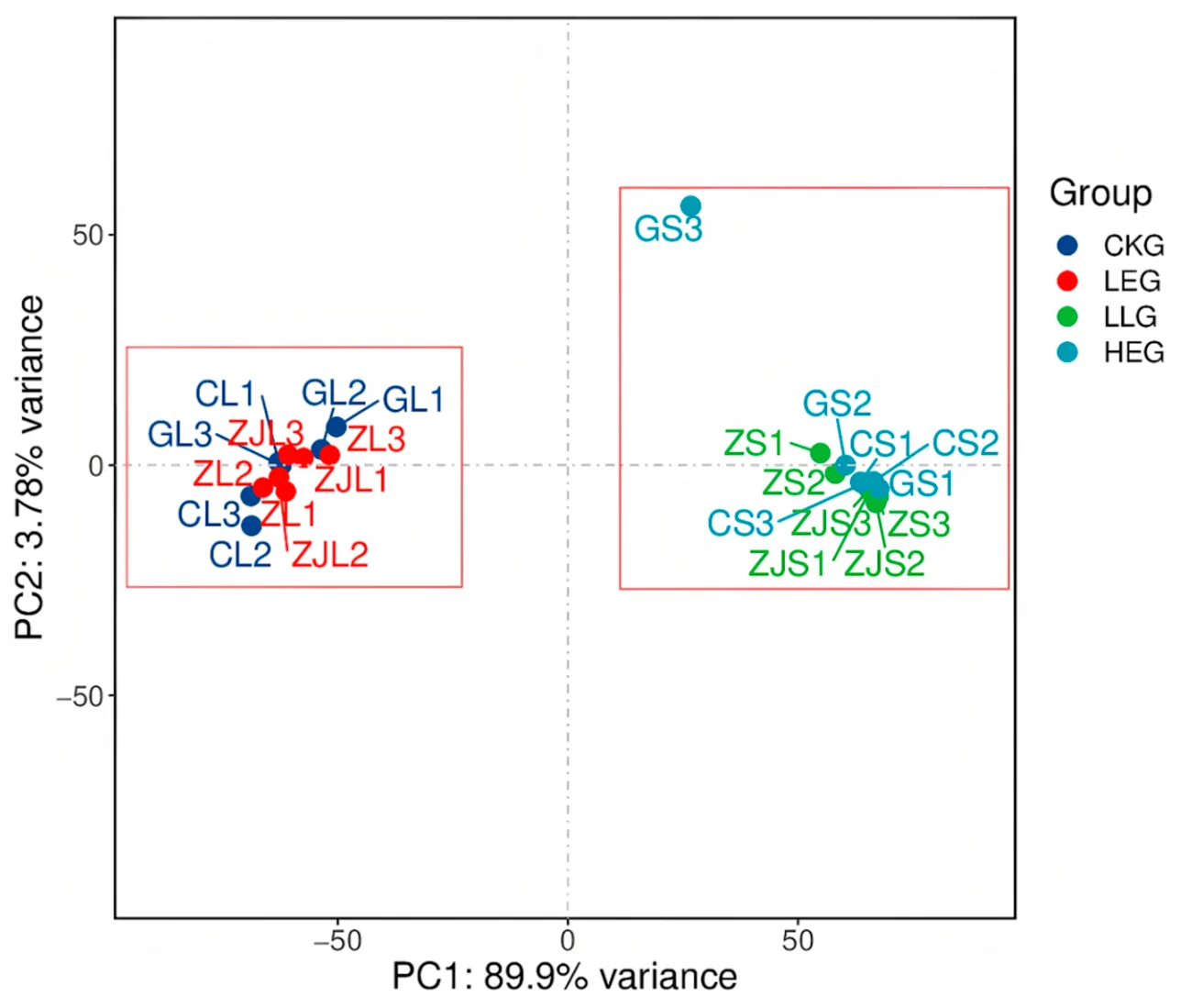
The bacterial PCoA analysis of rumen fluid at different fattening stages in yaks fed diets containing CNK (0.11 MJ/kg NEG), LEG (2.12 MJ/kg NEG), LLG (2.12 MJ/kg NEG + 0.02% Lactobacillus), and HEG (2.69 MJ/kg NEG).

To investigate Lactobacillus diets and rumen-specific genes involved in microbiota function and nutrient digestion, we characterized transcriptional profiles across different treatments of rumen tissues. Twenty-four yak rumen tissues from four groups were sequenced for transcriptomics. A total of 142.08 G of high-quality clean sequencing data were generated from all rumen tissue samples. Each sample yielded 5.52–6.87 G of clean reads, with Q30 scores ranging from 97.23% to 99.01%. These metrics collectively indicate excellent sequencing accuracy and are reliable for satisfying the quality requirements for downstream transcriptomic analyses. Principal component analysis (PCA) was performed to assess the impact of Lactobacillus intervention on differentially expressed genes in the rumen (Figure 9). The first principal component (PC1) explained 89.9% of the total variance, and the second (PC2) accounted for 3.78%, indicating that gene expression profiles were strongly driven by the dominant PC1 source of variation. Clear separation was observed between two major clusters: LEG and CKG clustered together, whereas LLG and HEG formed a distinct group, which suggested that both high-dose Lactobacillus supplementation and high-energy diet exerted convergent effects on rumen transcriptomic profiles.

**Figure 10:**
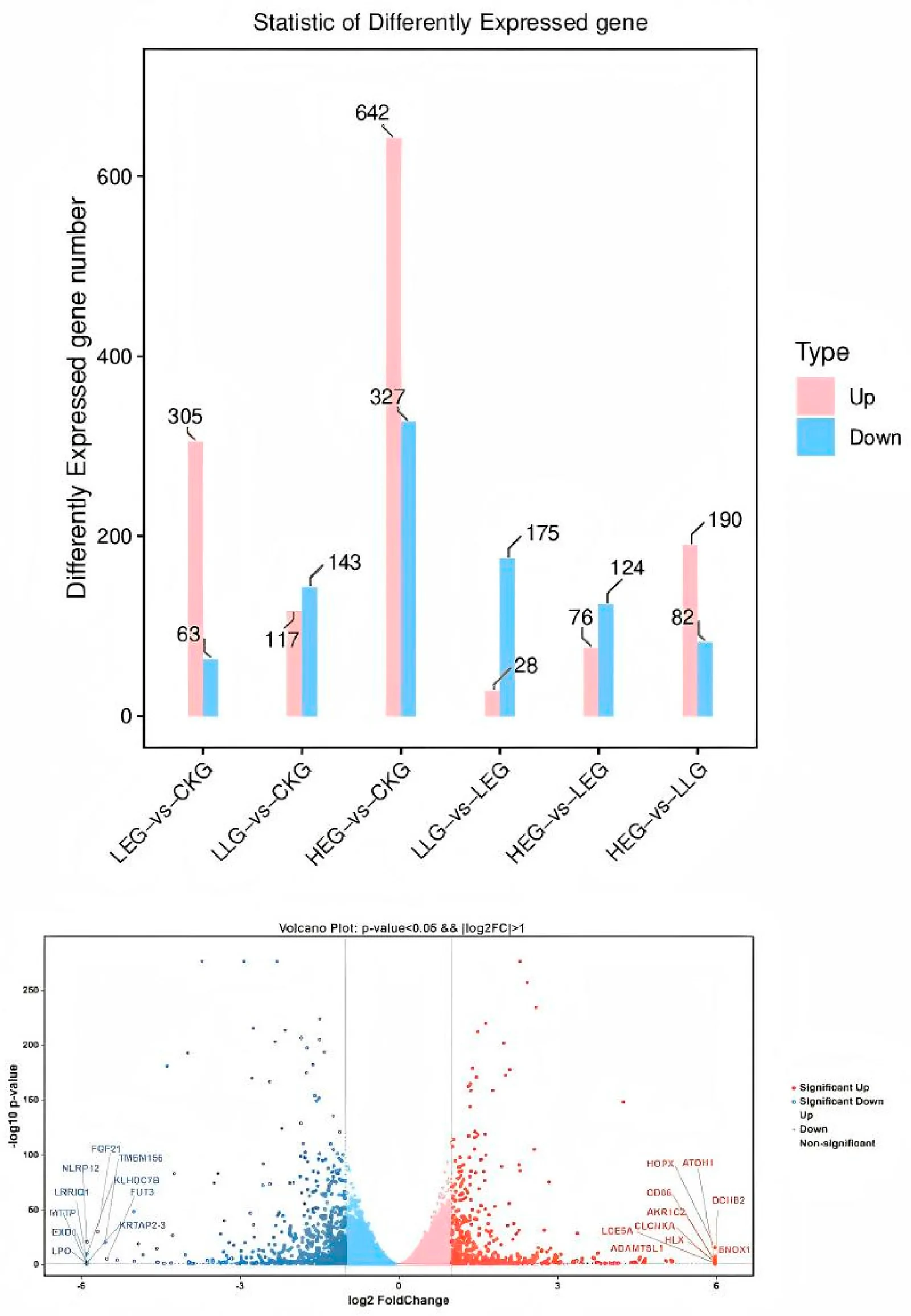
The volcano plot analysis for the identification of different expressed genes of rumen fluid at different fattening stages in yaks fed diets containing LEG (2.12 MJ/kg NEG) and LLG (2.12 MJ/kg NEG + 0.02% Lactobacillus). Note. Each point in the figure represents a sample; red indicates that the gene is expressed at high levels, and blue indicates lower expression.

**Figure 11:**
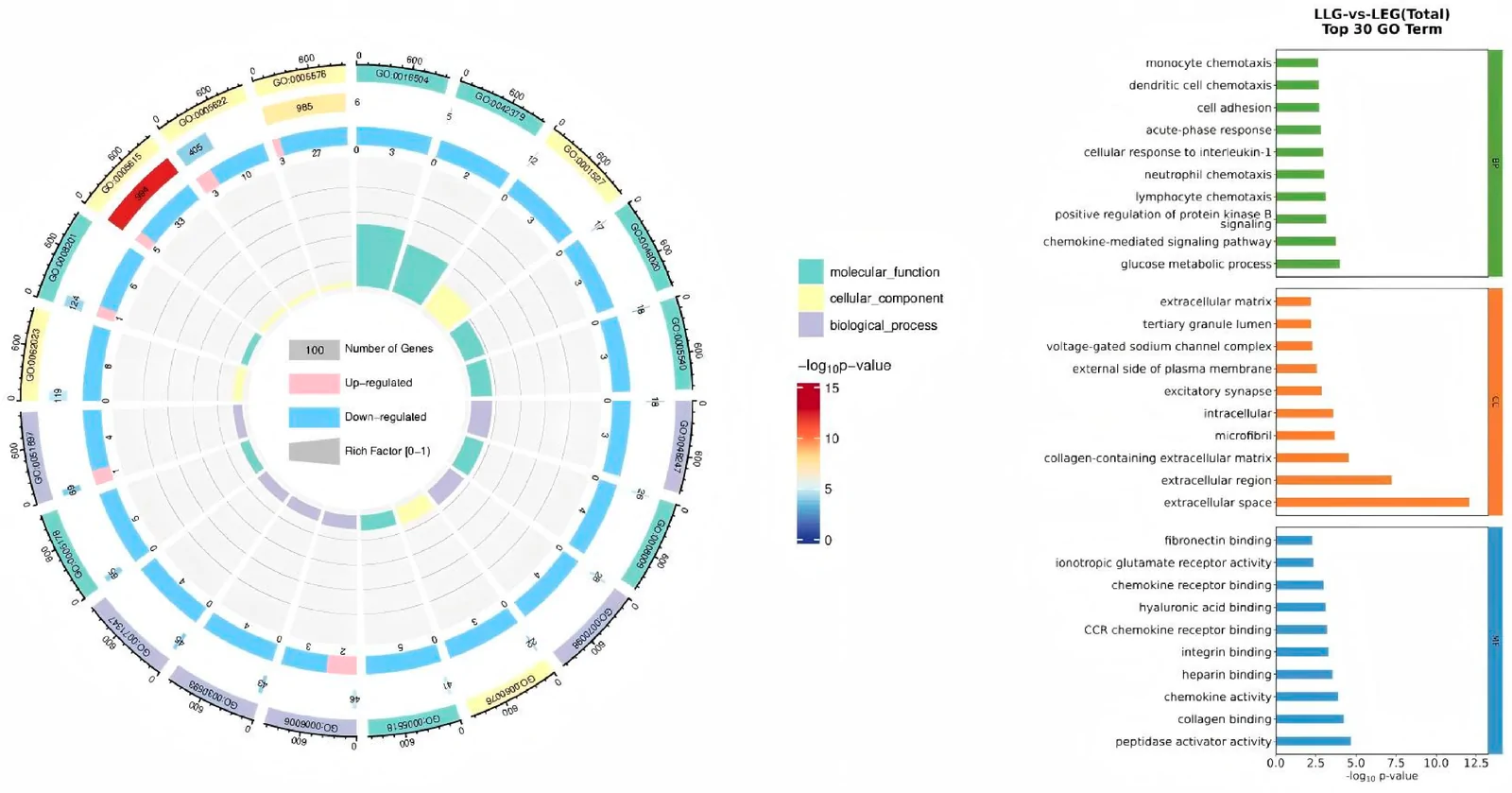
Predicted rumen microbial functions using enrichment GO analysis at different fattening stages in yaks fed diets containing CNK (0.11 MJ/kg NEG), LEG (2.12 MJ/kg NEG), LLG (2.12 MJ/kg NEG + 0.02% Lactobacillus), and HEG (2.69 MJ/kg NEG).

Pairwise analysis identified 368 differentially expressed genes (DEGs) between the LLG and LEG, including 305 upregulated and 63 downregulated genes in the LLG relative to the LEG (Figure 10). Concurrently, Gene Ontology (GO) enrichment analyses were performed on DEGs, and the results revealed that a total of 925 GO terms were identified, with 771 terms down-regulated and 154 terms up-regulated. We further employed the significance A/B test approach to screen the top 30 GO terms for subsequent analysis and annotated the proteins with cellular component (CC), molecular function (MF), and biological process (BP) terms (Figure 11). The results revealed that the most highly enriched terms associated with the differentially expressed proteins in the CC category were extracellular space (GO:0005615), collagen-containing extracellular matrix (GO:0062023), and extracellular matrix (GO:0031012). Other enriched terms in the MF category were collagen binding (GO:0005518), integrin binding (GO:0005178), and fibronectin binding (GO:0001968). The acute-phase response (GO:0006953) and chemokine-mediated signaling pathway (GO:0070098) were enriched in the BP category. These findings suggest that Lactobacillus supplementation may concurrently influence both nutrient metabolism and the physical/immune barrier functions in the rumen.

**Figure 12:**
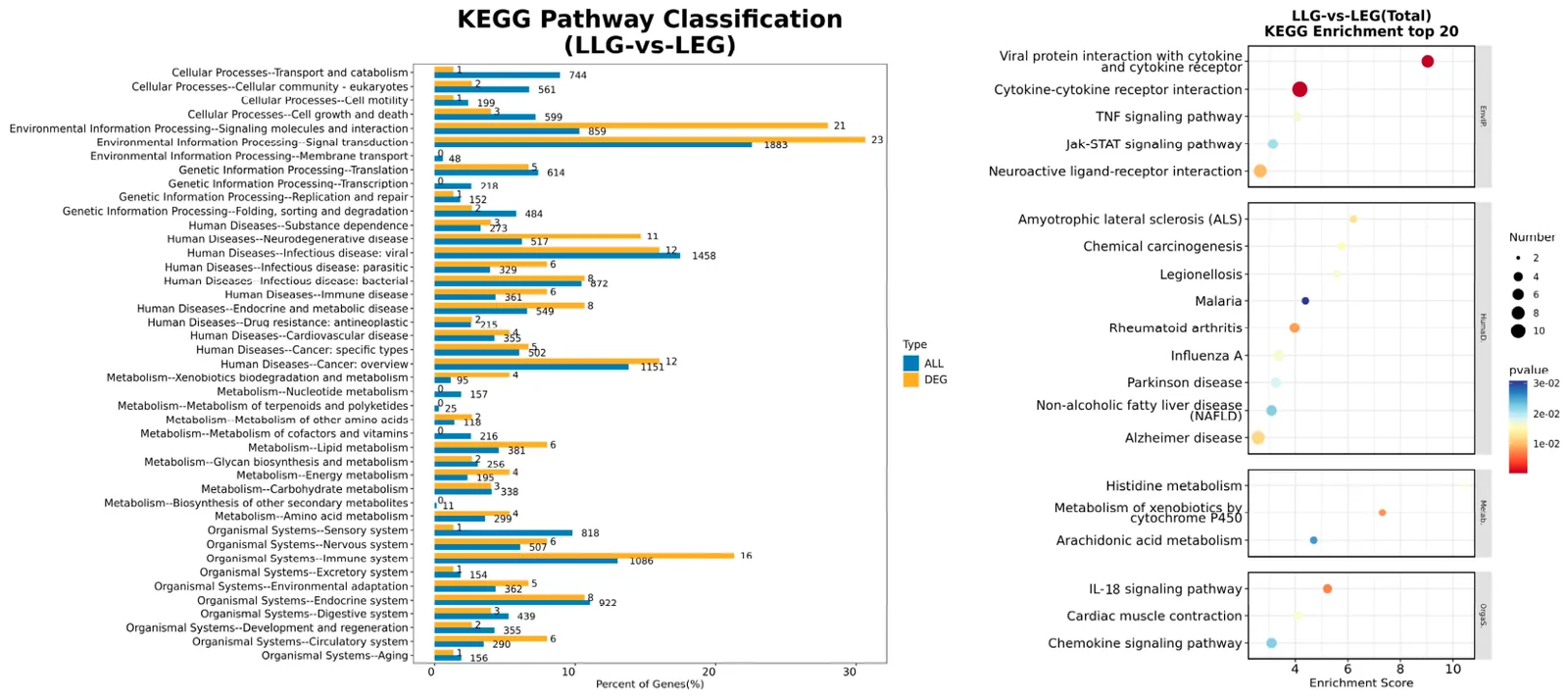
Predicted rumen microbial functions using enrichment KEGG analysis at different fattening stages in yaks fed diets containing CNK (0.11 MJ/kg NEG), LEG (2.12 MJ/kg NEG), LLG (2.12 MJ/kg NEG + 0.02% Lactobacillus), and HEG (2.69 MJ/kg NEG).

To identify key pathways related to digestive metabolism and barrier function, KEGG pathway enrichment analysis was performed on differentially expressed genes in rumen tissues between the LEG and LLG (Figure 12). Among 162 pathways analyzed, 39 were significantly enriched (*p* < 0.05). Several top enriched pathways are notably associated with immune and inflammatory regulation, including Cytokine–cytokine receptor interaction (ko04060), the IL-18 signaling pathway (ko05482), the TNF signaling pathway (ko04668), the Chemokine signaling pathway (ko04062), complement and coagulation cascades (ko04610), and the NOD-like receptor signaling pathway (ko04621). Pathways linked to nutrient metabolism were also enriched, such as histidine metabolism (ko00340), the metabolism of xenobiotics by cytochrome P450 (ko00980), and insulin resistance (ko04931). These KEGG results align with the GO enrichment findings, further supporting that Lactobacillus supplementation influences both rumen barrier integrity and metabolic processes.

**Figure 13:**
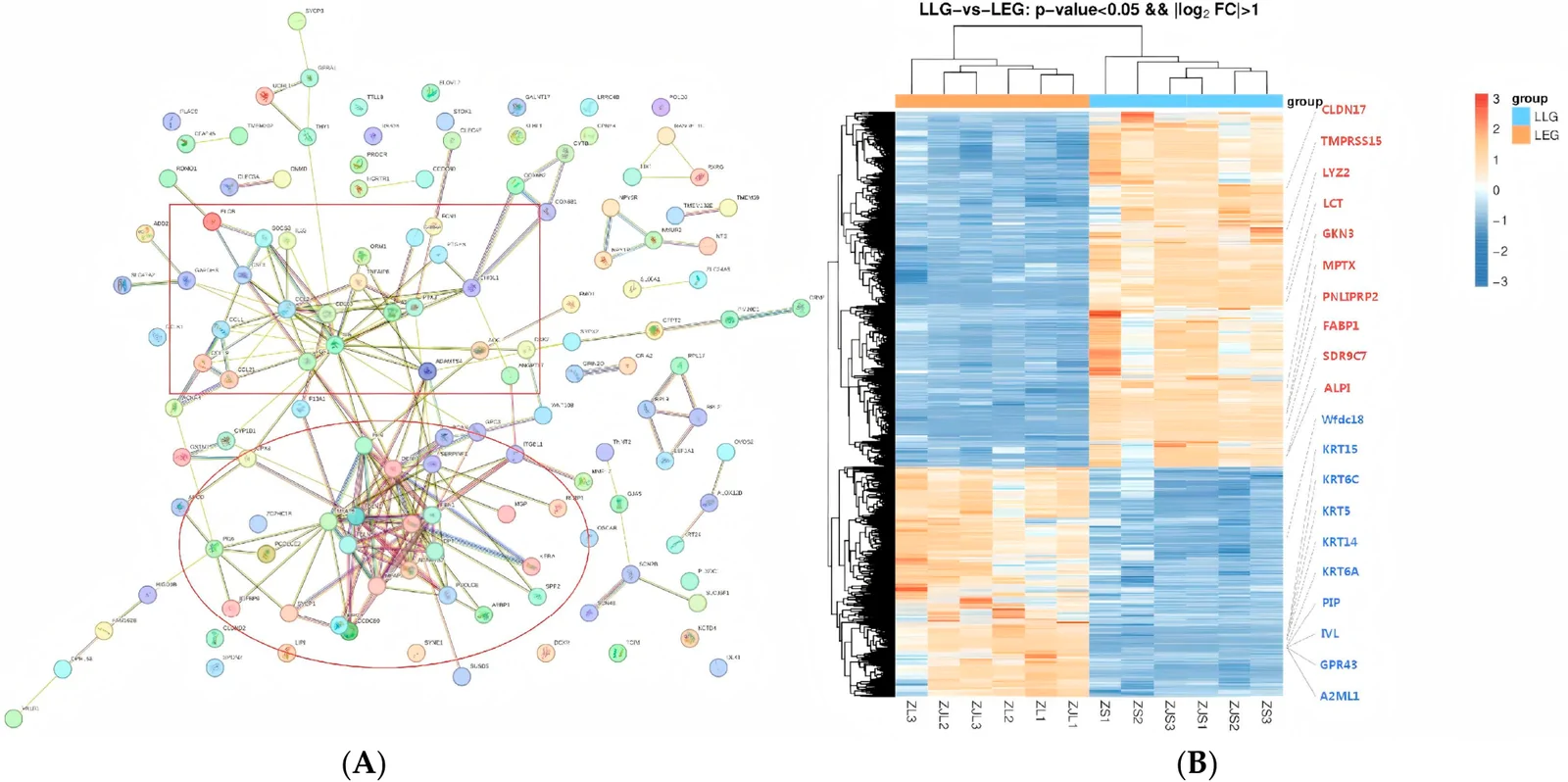
The differential abundance genes of rumen fluid in yaks during various fattening phases, fed diets supplemented with LEG (2.12 MJ/kg NEG) and LLG (2.12 MJ/kg NEG + 0.02% Lactobacillus) based on the STRING program. (**A**) Interaction network of candidate target genes, the rectangular boxes denote functional modules of genes associated with immune defense, whereas elliptical shapes represent functional modules of genes linked to nutrient metabolism; (**B**) The differential abundance genes of clustering analysis between LLG and LEG.

Potential interactions among the 368 DEGs were examined with STRING 12.0. The resulting network was highly connected and separated mainly into nutrient-metabolism and immune-defense modules (Figure 13). An A/B significance test was then used to prioritize enriched biological-process terms. Genes linked to enhanced digestion and metabolism included LCT, PNLIPRP2, FABP1, and ALPI, whereas LYZ2, GPR43, A2ML1, KRT5, KRT6A, and KRT14 were associated with mucosal barrier regulation and defense.

### 3.4. Multi-Omics Signature Integration Research for High-Concentrate Diets Mediated and Lactobacillus Intervention Treatment

**Figure 14:**
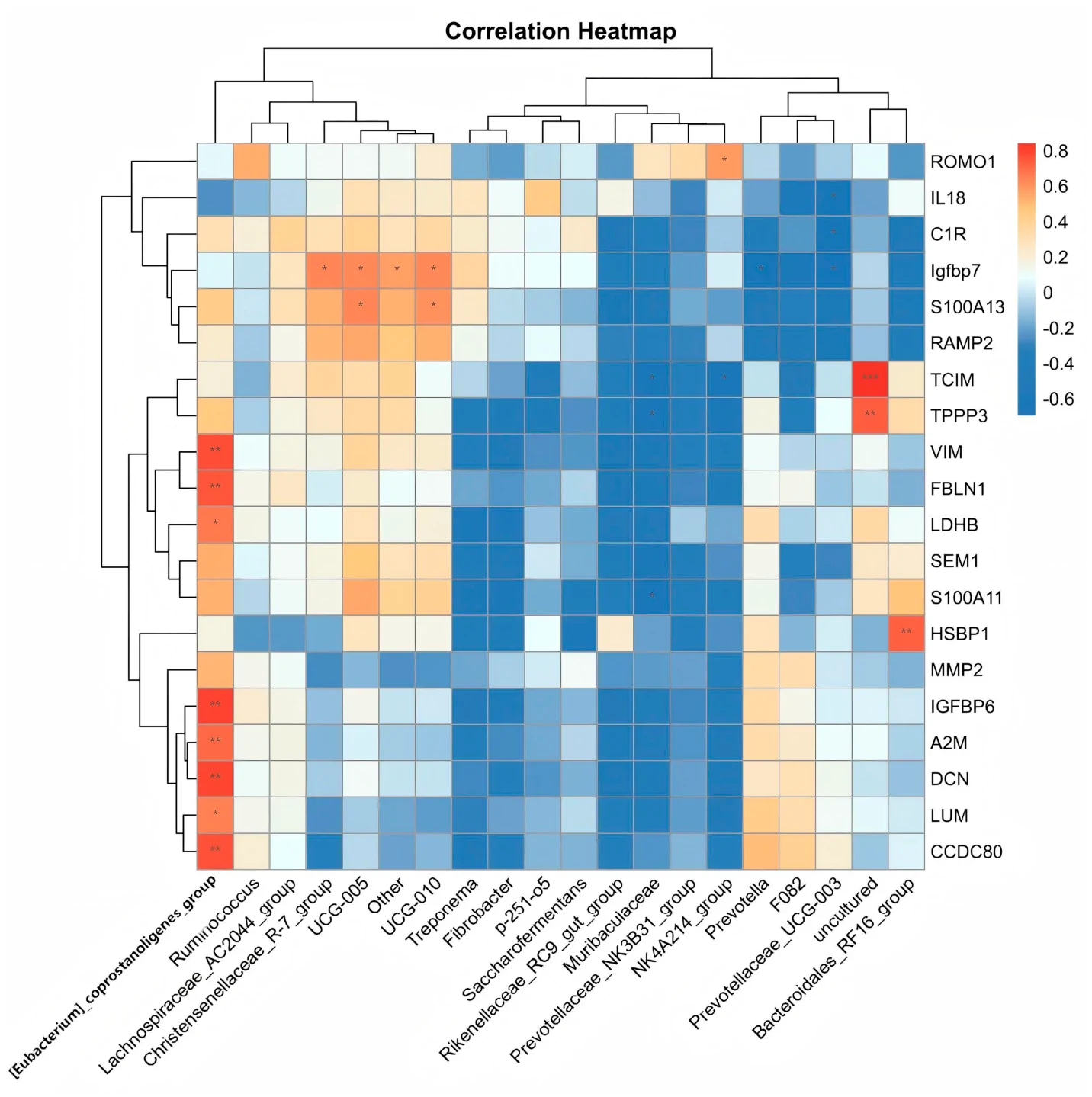
Correlation analysis between rumen microbiota and different expressed genes of rumen fluid in the yaks of the diet groups LEG (2.12 MJ/kg NEG) and LLG (2.12 MJ/kg NEG + 0.02% Lactobacillus). Note. Red represents a positive correlation, while blue represents a negative correlation. * significant correlation between LEG and LLG (*p* < 0.05), ** significant correlation between LEG and LLG (*p* < 0.01), and *** significant correlation between LEG and LLG (*p* < 0.001).

Spearman’s correlations were calculated to integrate rumen microbial features, nutrient digestibility, and host transcriptomic changes (Figure 14). Associations with |r| > 0.5 and *p* < 0.05 were considered significant. The correlation analysis between significantly enriched ruminal microbes and differentially expressed host genes revealed that Lactobacillus abundance was significantly positively correlated with the expression of IL-18, VIM, and LDHB (*p* < 0.05). Prevotella abundance showed significant negative correlations with S100A11 and CIR, while exhibiting a significant positive correlation with FABP1 (*p* < 0.05). Ruminococcus abundance was significantly positively associated with MMP2 and CLDN (*p* < 0.05). Collectively, these microbe–host gene associations suggest that Lactobacillus enrichment may reinforce rumen mucosal immunity, while coordinated modulation of Prevotella and Ruminococcus contributes to balanced mucosal homeostasis through complementary regulation of antimicrobial defense, inflammatory resolution, and epithelial barrier integrity. Integrated multi-omics correlation analysis of ruminal microbiota and host transcriptome revealed that Lactobacillus and their metabolic products act as signal initiators to activate the IL-1β signaling pathway, inducing the release of chemokines and recruiting immune cells such as monocytes and neutrophils, thereby enhancing mucosal immune responses. Meanwhile, Lactobacillus regulate the interaction between rumen microbiota and host genes, promoting the fermentation of structural carbohydrates in the rumen and driving the increase in the production of SCFAs such as propionic acid and isovaleric acid, thereby enhancing the efficiency of nutrient conversion. Therefore, we believe that the addition of Lactobacillus improves the digestive and absorptive efficiency of the rumen in yaks and strengthens the mucosal immune barrier function through multi-faceted regulation of microbiota–metabolism–immunity, providing a theoretical basis for the precise regulation of rumen health in yaks.

## 4. Discussion

Yak production constitutes the primary economic livelihood for pastoralists in plateau regions. To enhance production efficiency, livestock farmers commonly adopt high-energy or high-fat dietary regimens for yaks. However, mounting evidence indicates that such nutritional intensification imposes excessive gastrointestinal metabolic burden, thereby compromising animal health [28]. This study systematically investigated the regulatory effects of Lactobacillus intervention on rumen microbial homeostasis, nutrient digestion and barrier function-related genes in Pamir yaks under high-energy diet (HED) feeding during the cold season, which focused on clarifying how Lactobacillus modulates between ruminal microbiota remodeling and gene expression reprogramming in response to HED feeding and Lactobacillus intervention, and whether it can alleviate the adverse effects of high-concentrate diets on yak nutrient utilization and rumen health. Our research found that Lactobacillus intervention triggered a targeted restructuring of the yak rumen microbial community, characterized by a significant reduction in the relative abundance of Prevotellaceae and Ruminococcaceae, concomitant enrichment of fiber-degrading and SCFAs-producing bacteria, and a marked optimization of the dominant phylum-level architecture, notably an elevated Firmicutes-to-Bacteroidota (F/B) ratio. Lactobacillus supplements significantly enhanced ruminal fermentation efficiency, which elevated the concentrations of SCFAs and lowered the acetate-to-propionate (A/P) ratio, thereby counteracting the fermentation imbalance typically induced by high-concentrate diets. These improvements in ruminal metabolism directly translated to significantly higher apparent digestibility of dry matter, crude protein, and fibrous components (NDF, ADF, CF). Lactobacillus and its derived SCFAs functioned as signaling modulators, which activated pivotal host immune pathways (IL-17, TNF-α), upregulated the expression of immune-related genes (IL-18, VIM, LDHB), and strengthened the rumen mucosal barrier. This intervention coordinated the expression of key nutrient metabolism genes (LCT, PNLIPRP2, FABP1) to promote overall rumen digestive and absorptive capacity.

Rumen microorganisms play a central role in host energy supply, SCFAs production, and immune regulation [29]. Previous studies have demonstrated that approximately 70–80% of the energy required by yaks is derived from rumen microbial fermentation [30]. Disruption of rumen structure or function can therefore reduce feed efficiency, productivity, and health [31]. Lactobacillus is recognized as a natural alternative to antibiotics and plays an important role in the process of rumen fermentation, producing organic acids during host metabolism, as well as inhibiting the growth of undesirable bacteria [32]. Yaks receiving Lactobacillus intervention showed faster FBW and FSW, especially after adaptation to the re-resistive colonization of intestinal microorganisms during the initial fattening stage. This indicates that Lactobacillus exhibits significant potential as an effective feed additive for re-engineering microbiota function and metabolism and enhancing production efficiency, findings consistent with earlier studies. SCFAs are primarily derived from carbohydrate hydrolysis mediated by ruminal microbes. During the fattening period, with the increase in dietary energy levels and the corresponding rise in the proportion of dietary carbohydrates, the concentrations of acetic acid and propionic acid were significantly elevated. This phenomenon is primarily attributed to acetic acid and propionic acid, and acetic acid acts as a precursor for lipogenesis in ruminants, while propionic acid plays a critical role in hepatic glycogen synthesis [33]. However, the concurrent significant increase in the acetic acid/propionic acid ratio suggests that this elevation in SCFAs is achieved at the expense of exacerbated fermentation imbalance and disruption of the microbial community structure. Notably, Lactobacillus intervention can reduce the acetic acid/propionic acid ratio while maintaining acetic acid and propionic acid at relatively high levels, thereby enhancing rumen fermentation efficiency and stabilizing the microbial community structure. This finding is further validated by the significant improvement in the apparent digestibility of DM, CP, and CF following Lactobacillus supplementation. Collectively, these results indicate that Lactobacillus supplementation during fattening with high-concentrate diets can enhance rumen fermentation function by modulating the rumen microbial community and its structure, thereby regulating host functional homeostasis and facilitating healthy livestock production.

The rumen microbiota plays a pivotal role in the digestive system of ruminants, including cattle, sheep, and other similar species [34]. The microbiota is responsible for the conversion of plant fiber and other indigestible substances into absorbable nutrients, and Lactobacillus could maintain rumen health and show beneficial effects on growth, potential, and nutrient digestibility in yaks [35]. The current study found that the number of OTUs was significantly reduced under Lactobacillus intervention during all stages of fattening, which may be attributed to intense competition for nutrients and space between exogenous Lactobacillus and the host microbiota, which changed the rumen microenvironment to promote more efficient functional collaboration. These results suggest that a high-energy diet may compromise the stability of the microbial community structure and its uniform distribution, leading to reduced nutrient utilization efficiency. Additionally, we observed that following an initial two-month adaptation period, the OTU count in the LLG began to recover and exhibited an upward trend. This indicates that after undergoing adaptive colonization, Lactobacillus reshapes the microbial community toward functional adaptation by promoting the synergistic growth of beneficial bacterial groups. Integrating the results of α diversity analysis, we postulate that the supplementation of lactic acid bacteria enhances the microbiota’s capacity for fiber degradation and short-chain fatty acid synthesis, while also inhibiting the ability of fat deposition. Moreover, the presence of a high-energy diet leads to the over-proliferation of Ruminococcaceae, thereby causing negative effects on weight gain. Further diversity analysis demonstrated that Lactobacillus supplementation slightly reduced species richness, which significantly enhanced community evenness. This suggests that high-energy diets may drive the over-proliferation of a limited number of starch-degrading microorganisms. Lactobacillus intervention facilitates the selection of dominant genera that contribute to the optimization of host barrier function, thereby enhancing community stability, enabling efficient synergistic nutrient decomposition and metabolism, and ultimately promoting animal health, which was consistent with the results of PCoA. Lactobacillus intervention significantly elevated the Firmicutes-to-Bacteroidetes (F/B) ratio. While the implications of the F/B ratio shifts remain contentious in obesity research, an increased F/B ratio in ruminants is frequently linked to enhanced fiber degradation capacity and SCFAs production [36]. The findings of this study align with this notion, indicating that Lactobacillus supplementation may have enriched Firmicutes taxa associated with fiber decomposition. Lactobacillus intervention led to a significant decrease in the relative abundance of the Prevotellaceae family and its subordinates UCG-003 and UCG-004. Meanwhile, the abundance of families related to functions such as protein metabolism, mucus degradation, or butyric acid production, such as Peptostreptococcaceae, Muribaculaceae, and Butyricicoccaceae, showed an upward trend. This finding is consistent with the functional prediction analysis of PICRUSt2, which indicated that after Lactobacillus intervention, the “carbohydrate transport and metabolism” function was enhanced, while the “cell wall/membrane/envelope biosynthesis” function was weakened. This suggests that Lactobacillus can substantially enhance the intestinal capacity for the decomposition and absorption of dietary carbohydrates by regulating the microbial community.

Rumen epithelial gene expression provides a direct view of changes in barrier activity. Correlating host transcripts with microbial features therefore helps clarify how probiotic supplementation affects microbiota–host interactions. Lactobacillus can buffer the rumen against adverse feeding conditions [37], and previous studies have associated supplementation with higher microbial metabolic activity, greater nutrient availability, and improved yak production [38,39,40]. Our results showed Lactobacillus supplementation induced significant rumen transcriptomic reprogramming, primarily enhancing nutrient metabolic capacity and reinforcing mucosal immune defense. These changes are closely intertwined with alterations in the ruminal microbial community and its metabolic outputs related to SCFAs. Lactobacillus intervention significantly remodeled extracellular environment and cell surface, which remodeling is likely crucial for the integrity of the mucosal barrier to enhance signaling capabilities and molecular interactions at the host–microbe interface, which remodeling is likely crucial for the integrity of the mucosal barrier. Most notably, KEGG pathway analysis corroborated and expanded upon the findings from GO analysis. The enrichment between the metabolic efficiency pathway of the “glucose metabolic process” and the immune functions pathway of “IL-17 signaling pathway” and “TNF signaling pathway” revealed a tightly coordinated crosstalk, which underscored Lactobacillus as a probiotic, not only modulating host immune homeostasis but also counteracting the metabolic stress induced by high-concentrate feeding. The integration of transcriptome and microbiome through correlation analysis provided the crucial link between the microbial community, SCFAs, and host transcriptional response. The positive correlations between SCFAs and nutrient digestibility directly benefit Lactobacillus-induced microbial function to enhance rumen fermentation efficiency. More importantly, the positive correlation between Lactobacillus abundance and the expression of immune-related genes (IL-18, VIM) and metabolic genes (LDHB) suggests a direct or indirect role for this bacterium in co-regulating these processes. The contrasting correlations of Prevotella and Ruminococcus with different host genes (S100A11, FABP1, MMP2, CLDN) imply a complex, perhaps complementary, role for different microbial taxa in maintaining mucosal homeostasis by rumen barrier integrity.

## 5. Conclusions

This study systematically investigated the dynamic regulation of rumen microbial diversity by Lactobacillus via resistant colonization under the cold-season high-concentrate fattening regime. Specifically, Lactobacillus reduced the abundance of Prevotella and Ruminococcus, elevated the Firmicutes-to-Bacteroidetes ratio, enhanced the relative abundance of functional bacterial groups associated with fiber degradation and short-chain fatty acid (SCFA) synthesis, and mitigated the potential dysbiosis of the microbial community induced by high-energy diets. Lactobacillus intervention significantly decreased the acetic acid/propionic acid ratio while maintaining the concentrations of acetic acid, propionic acid, and butyric acid in the rumen, thereby improving rumen fermentation balance. Additionally, it increased the apparent digestibility of dry matter, crude fiber, and crude protein to promote efficient nutrient absorption. Concurrently, Lactobacillus activated signaling pathways such as IL-17 and TNF, upregulated the expression of genes related to nutrient metabolism and immunity, and strengthened rumen barrier function. Collectively, these findings provide a probiotic-based regulatory strategy for the intensive fattening of yaks in alpine pastoral regions.

## Figures and Tables

**Table 1:** The feed ingredient and nutrient composition of Yak diets containing CNK (0.11 MJ/kg NEG), LEG (2.12 MJ/kg NEG), and HEG (2.69 MJ/kg NEG).

Composition (%)	CKG	LEG	HEG
Maize	18.7	29.8	34.4
Cottonseed meal	-	4.6	5
Soybean meal	-	6.0	6.5
Soybean oil	-	-	0.6
Wheat bran	3.8	5.8	1
Molasses	-	1.3	-
Premix ^②^	2.5	2.5	2.5
Wheat straw	71.6	50	50
Cottonseed hulls	3.4	-	-
Total	100	100	100
Nutritional composition ^③^			
CP (%)	5.26	9.85	10.92
NEG (MJ/Kg) ^①^	0.11	2.12	2.69
ADF (%)	8.49	5.18	4.51
NDF (%)	44.41	43.42	41.84
Ca	1.61	1.62	1.59
P	0.44	0.55	0.54

Note: ① NEG was a calculated value (NRC, 2007), while others were measured values. ② The premix provided the following per kg of diet: Fe 2500 mg, Zn 1000 mg, Cu 1000 mg, Mn 1000 mg, Se 7.5 mg, I 20 mg, Vitamin A 300,000 IU, Vitamin D 5000 IU, Vitamin E 4000 IU. ③ CP was crude protein, NEG was net energy for gain, ADF was acid detergent fiber, DNF was neutral detergent fiber, CKG was the control feeding diet group, LEG was the normal-energy diet group, and HEG was the high-energy diet group.

**Table 2:** The body weight (BW) and average daily gain (ADG) when feeding Yak diets containing CNK (0.11 MJ/kg NEG), LEG (2.12 MJ/kg NEG), LLG (2.12 MJ/kg NEG + 0.02% Lactobacillus), and HEG (2.69 MJ/kg NEG).

	CKG	LEG	LLG	HEG
Initial body weight (IBW)/kg	252.20 ± 9.60	245.35 ± 10.55	252.10 ± 12.50	251.30 ± 17.25
Final body weight (FBW)/kg	276.15 ± 13.25 d	293.35 ± 19.25 c	355.45 ± 22.40 b	370.85 ± 36.10 a
1st Fattening stage weight (FSW1)/kg	9.80 ± 1.67 c	12.80 ± 2.06 b	11.90 ± 1.62 bc	16.10 ± 1.55 a
2nd Fattening stage weight (FSW2)/kg	7.40 ± 2.49 c	11.40 ±1.19 b	15.20 ± 2.23 b	20.40 ± 1.73 a
3rd Fattening stage weight (FSW3)/kg	2.15 ± 1.83 c	13.10 ± 3.05 b	30.70 ± 2.54 a	33.50 ± 5.98 a
4th Fattening stage weight (FSW4)/kg	4.05 ± 3.16 c	12.70 ± 3.86 b	45.10 ± 5.22 a	49.60 ± 3.17 a
Total average daily gain (TADG)/g/day	140.47 ± 27.22 c	415.47 ± 33.25 b	651.77 ± 70.71 ab	827.43 ± 102.74 a

Note. Different letters represent significant differences (*p* < 0.05). Values are presented as mean ± SEM (n = 30). Homogeneity of variances was confirmed by Bartlett’s test (*p* > 0.05).

**Table 3:** Lactobacillus intervention on apparent nutrient digestibility for the yak diets of LEG (2.12 MJ/kg NEG) and LLG (2.12 MJ/kg NEG + 0.02% Lactobacillus) (g/d, %).

Nutritional Composition (%)	LLG	LEG
DM	78.70 ± 0.82 a	70.65 ± 0.28 b
CP	49.97 ± 0.68 a	41.45 ± 1.07 b
NDF	79.08 ± 0.63 a	71.83 ± 0.92 b
ADF	80.03 ± 0.68 a	70.06 ± 0.44 b
CF	78.04 ± 0.69 a	70.38 ± 0.85 b
EE	74.35 ± 0.38	72.73 ± 0.83
Ash	67.94 ± 0.63	64.56 ± 1.05

Note. Different letters represent significant differences (*p* < 0.05). Values are presented as mean ± SEM (n = 3). Homogeneity of variances was confirmed by Bartlett’s test (*p* > 0.05). DM was dry matter, CP was crude protein, NDF was neutral detergent fiber, ADF was acid detergent fiber, CF was crude fiber, EE was ether extract.

**Table 4:** The alpha diversity of bacterial communities when feeding Yaks different diets containing CNK (0.11 MJ/kg NEG), LEG (2.12 MJ/kg NEG), LLG (2.12 MJ/kg NEG + 0.02% Lactobacillus), and HEG (2.69 MJ/kg NEG).

Group		Shannon	Simpson	Ace	Chao1
1st month	CKG1	1.03 ± 0.17 b	0.56 ± 0.11 a	12.82 ± 6.23	12.34 ± 2.25
	LEG1	0.85 ± 0.09 c	0.58 ± 0.07 a	13.39 ± 5.03	13.51 ± 2.35
	LLG1	1.12 ± 0.07 a	0.64 ± 0.03 a	12.73 ± 2.37	12.01 ± 1.79
	HEG1	0.75 ± 0.15 d	0.42 ± 0.06 b	15.84 ± 3.61	13.17 ± 1.47
2nd month	CKG2	0.97 ± 0.16 a	0.49 ± 0.09 c	12.29 ± 6.08	12.67 ± 1.03
	LEG2	0.87 ± 0.08 ab	0.56 ± 0.04 b	12.99 ± 1.04	13.52 ± 0.84
	LLG2	0.99 ± 0.16 a	0.61 ± 0.17 a	10.57 ± 5.69	12.12 ± 2.03
	HEG2	0.82 ± 0.07 b	0.48 ± 0.03 c	13.94 ± 6.24	13.21 ± 1.57
3rd month	CKG3	1.29 ± 0.18 a	0.56 ± 0.06 a	13.78 ± 4.05 c	12.29 ± 4.26 b
	LEG3	0.89 ± 0.16 b	0.53 ± 0.13 a	15.52 ± 2.78 b	13.25 ± 0.88 b
	LLG3	1.24 ± 0.06 a	0.58 ± 0.04 a	13.62 ± 2.31 c	11.25 ± 1.17 b
	HEG3	0.78 ± 0.09 c	0.39 ± 0.04 b	23.77 ± 0.91 a	23.67 ± 0.82 b
4th month	CKG4	0.99 ± 0.15 a	0.66 ± 0.09 a	12.56 ± 3.91 c	13.73 ± 3.84 b
	LEG4	0.86 ± 0.09 b	0.52 ± 0.07 bc	14.41 ± 6.18 b	15.64 ± 1.18 a
	LLG4	1.06 ± 0.08 a	0.57 ± 0.05 b	11.72 ± 1.93 c	11.17 ± 1.72 b
	HEG4	0.83 ± 0.06 b	0.47 ± 0.02 c	16.91 ± 3.45 a	15.75 ± 1.61 a

Note. Different letters represent significant differences (*p* < 0.05). Values are presented as mean ± SEM (n = 6). Homogeneity of variances was confirmed by Bartlett’s test (*p* > 0.05).

## Data Availability

The datasets presented in this study can be found in online repositories. All raw sequence data were deposited in the NCBI Sequence Read Archive database under accession number PRJNA1233467.
